# Spectral splitting photovoltaics using perovskite and wideband dye-sensitized solar cells

**DOI:** 10.1038/ncomms9834

**Published:** 2015-11-05

**Authors:** Takumi Kinoshita, Kazuteru Nonomura, Nam Joong Jeon, Fabrizio Giordano, Antonio Abate, Satoshi Uchida, Takaya Kubo, Sang Il Seok, Mohammad Khaja Nazeeruddin, Anders Hagfeldt, Michael Grätzel, Hiroshi Segawa

**Affiliations:** 1 Research Center for Advanced Science and Technology (RCAST), The University of Tokyo, 4-6-1, Komaba, Meguro-ku, Tokyo 153-8904, Japan; 2 Department of Chemistry and Chemical Engineering, Laboratory of Photomolecular Science, Swiss Federal Institute of Technology, Station 6, CH-1015 Lausanne, Switzerland; 3 Division of Advanced Materials, Korea Research Institute of Chemical Technology, 141 Gajeong-Ro, Yuseong-Gu, Daejeon 305-600, Korea; 4 Department of Chemistry and Chemical Engineering, Laboratory of Photonics and Interfaces, Swiss Federal Institute of Technology, Station 6, CH-1015 Lausanne, Switzerland; 5 Komaba Organization for Educational Excellence (KOMEX), The University of Tokyo, Komaba 3-8-1, Meguro-ku, Tokyo 153-8902, Japan; 6 School of Energy and Chemical Engineering, Ulsan National Institute of Science and Technology (UNIST), 50 UNIST-gil, Eonyang-eup, Ulju-gun, Ulsan 689-798, Korea

## Abstract

The extension of the light absorption of photovoltaics into the near-infrared region is important to increase the energy conversion efficiency. Although the progress of the lead halide perovskite solar cells is remarkable, and high conversion efficiency of >20% has been reached, their absorption limit on the long-wavelength side is ∼800 nm. To further enhance the conversion efficiency of perovskite-based photovoltaics, a hybridized system with near-infrared photovoltaics is a useful approach. Here we report a panchromatic sensitizer, coded DX3, that exhibits a broad response into the near-infrared, up to ∼1100 nm, and a photocurrent density exceeding 30 mA cm^−2^ in simulated air mass 1.5 standard solar radiation. Using the DX3-based dye-sensitized solar cell in conjunction with a perovskite cell that harvests visible light, the hybridized mesoscopic photovoltaics achieved a conversion efficiency of 21.5% using a system of spectral splitting.

## Abstract (web summary)

Lead halide perovskite solar cells have a limited spectral response in the near infrared. Here, the authors present ruthenium dyes with extended near infrared absorbance and combine perovskite and dye-based solar cells by spectral splitting to obtain broadband, 21.5% efficient solar cells.

## Graphical abstract

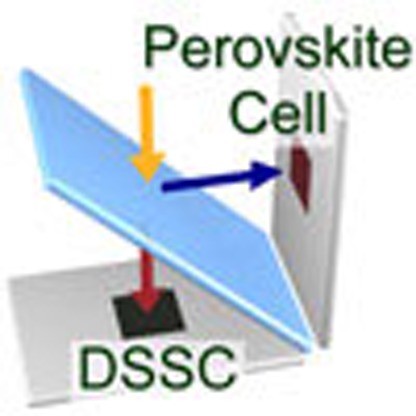

Dye-sensitized solar cells^1^,^2^,^3^ (DSSCs) and organic lead halide perovskite solar cells^4^,^5^,^6^,^7^,^8^,^9^ (PSCs) have attracted significant attention as next-generation low-cost photovoltaics because of the potential widespread applications^10^. Since the mesoscopic photovoltaics can be produced by simple solution processing, the technology shows potential for the large-scale mass production of solar cells. Furthermore, the power conversion efficiencies (PCEs) of lead halide PSCs have increased steeply over the past few years. However, the absorption range of lead halide PSCs is still limited to the visible region. In general, the PCE of a photovoltaic is limited by a voltage loss in the shorter wavelength region and an optical absorption loss in the longer wavelength region^11^,^12^. To reduce the energy losses, a multijunction architecture is effective. Although many inorganic semiconductor multijunction devices employing tandem^13^ or spectral splitting^14^,^15^,^16^ systems have been developed, their application is still limited because of problems in the production processes and total manufacturing costs. In contrast, several multijunction photovoltaics employing DSSCs^17^,^18^ or organic thin film^19^,^20^ are expected to be easier to produce, but their PCE currently remains at a relatively low level of ∼10–12%. To improve the PCE of multijunction organic cells, two independent types of solar cells should be assembled, one showing a high photovoltage and the other a large response in the near-infrared (NIR) region. Although several trials for the realization of the NIR photovoltaic cells such as DSSC using osmium sensitizers^21^,^22^,^23^,^24^ or ruthenium sensitizers with large π-conjugated ligands^25^,^26^, organic thin-film solar cells^27^ and tin halide PSCs^28^,^29^ have been reported, the device performance remains low. Recently, DSSCs using a phosphine-coordinated ruthenium sensitizer (DX1), which exhibit a direct singlet-to-triplet transition in the NIR region, have been developed to yield a panchromatic photoresponse^30^. A mechanically stacked tandem DSSC comprises DX1 as the NIR sensitizer for the bottom cell and N719 as a visible sensitizer for the top cell was reported to have a PCE of 12% under reduced sunlight^30^. Using a PSC as the visible-light-absorbing cell should substantially increase the PCE. However, the NIR response of DX1 is insufficient for such a combination. Here, we present a molecular design for the panchromatic sensitizer and the development of a PSC/DSSC hybridized device by a spectral splitting system. The panchromatic ruthenium complexes DX2 (*trans*-dichloro-(metyldiphenylphosphine)-(2,2′;6′,2′′-terpyridyl-4,4′,4′′-tricarboxylic acid-4-methyl ester)ruthenium(II)) and DX3 (*trans*-dichloro-(dimetylphenylphosphine)-(2,2′;6′,2′′-terpyridyl-4,4′,4′′-tricarboxylic acid-4-methyl ester)ruthenium(II)) are engineered on the molecular level to increase the spin–orbit interactions, enhancing the direct singlet-to-triplet transition and extending its light-harvesting capacity further into the NIR region than that of DX1 with the tuning of triplet excited state levels by the replacement of the phosphine ligands. By associating a DX3-based DSSC with a PSC with an 18.4% PCE and negligible hysteresis, we achieved a PCE of 21.5%.

## Results

### Properties of the panchromatic sensitizer

**Figure 1:**
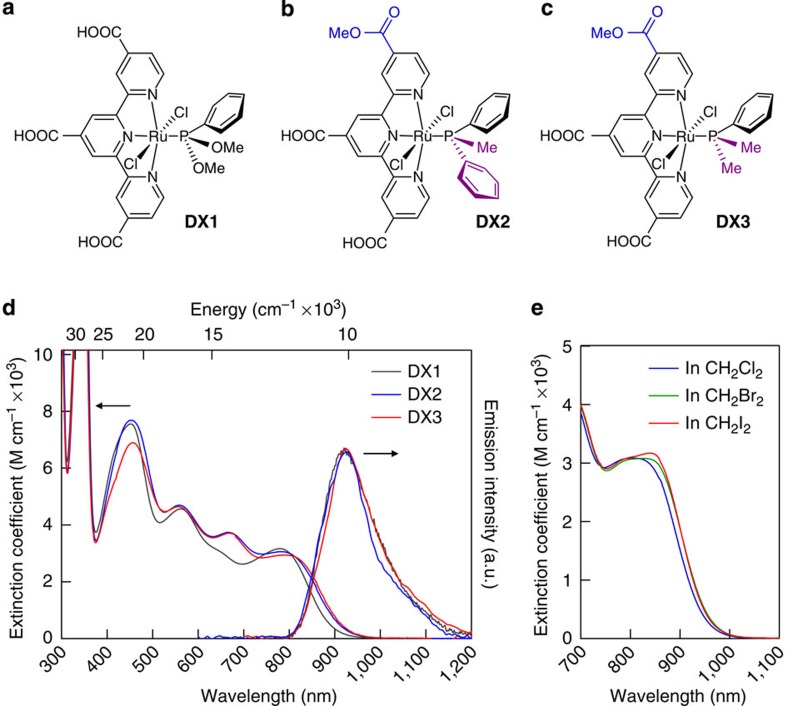
Chemical structure and absorption spectra of DX1, DX2 and DX3. (**a**–**c**) Chemical structure of DX1, DX2 and DX3, respectively. A carboxy group of DX1 is replaced with a methyl ester group (blue part in DX2 and DX3) for the improvement of solubility in organic solvents. Bulky substituents (purple part) in the phosphine ligand are introduced to control the energy level of the excited triplet states. (**d**) Absorption and emission spectra of DX1, DX2 and DX3 in DMF solution. The emission spectra were measured at 77 K. (**e**) Absorption spectra of esterified DX3 in methylene dihalide (CH_2_X_2_:X=Cl, Br, I) solutions at 298 K. The lowest absorption peak was enhanced in the heavier halogenated solvent.

**Figure 2:**
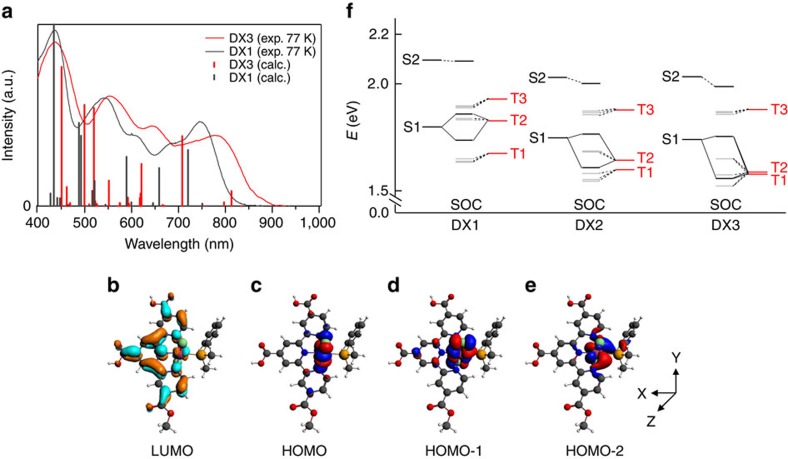
Calculated absorption spectra and excitated states energy diagram of panchromatic sensitizers. (**a**) Low-temperature absorption spectra and calculated absorption spectra of DX1 and DX3 including spin–orbital coupling effects by SOC-TD-DFT. (**b**–**e**) Molecular orbitals of DX3. Highest occupied (HOMO) and lowest unoccupied (LUMO) molecular orbitals of DX3 as calculated using scalar relativistic DFT at the M06/TZP with ADF program. (**f**) Excitated states energy diagram that showing SOC of DX1, DX2 and DX3.

The chemical structures of DX1, DX2 and DX3 are shown in Fig. 1a–c, respectively. DX2 and DX3 feature a methyldiphenylphosphine and dimethylphenylphosphine ligand, respectively, with a weaker ligand field than that of the dimethoxyphenylphosphine employed in the previously reported DX1 sensitizer^23^. Since one of the three carboxyl groups on the tricarboxy-terpyridyl (tcterpy) ligand was esterified, the solubility of DX2 and DX3 was improved, as compared with DX1. The absorption spectra of DX1, DX2 and DX3 in *N*,*N*-dimethylformamide (DMF) solution are shown in Fig. 1d. DX2 and DX3 showed an intense ultraviolet absorption band at 318 nm that was assigned to the ligand-centred π–π* transition of the tricarboxy-terpyridyl ligand and broad absorption bands in the visible region that were assigned to metal-to-ligand charge-transfer transitions. DX2 and DX3 have absorption spectra similar to that of DX1 in the visible region. A notable difference is that the tail of the NIR band in DX3 is extended by 50 nm to a longer wavelength, as compared with DX1, especially DX3 showed the longest wavelength absorption up to 1,000 nm in these sensitizers. The calculated and low-temperature absorption spectra of DX1 and DX3 in EtOH/2-methyl-THF are shown in Fig. 2a. The absorption spectrum of DX3 at 77 K becomes sharper, and a broad absorption band featuring a peak with a shoulder is observed at ∼790 nm. A similar absorption band structure was observed in the di-*tert*-butyl ester derivative of DX3 at 77 K in toluene (Supplementary Fig. 1). The absorption intensity in the NIR region of the esterified complex in a methylene dihalide solvent increases in proportion to the atomic weight of the halogen atom (Fig. 1e). These changes are induced by an external heavy atom spin–orbital coupling effect^31^. The effect of external heavy atom was small since the internal heavy atom effect by ruthenium centre acted strongly. We calculated the absorption spectra of DX1 and DX3 using time-dependent density functional theory (TD-DFT) based on a two-component relativistic Hamiltonian including spin–orbit coupling (SOC) self-consistent^32^ with an M06 functional^33^ (Supplementary Methods). From the calculations, DX3 is expected to show a substantial red-shifted absorption band as compared with DX1, which is borne out by our experimental observations (Fig. 2a). The part of the calculated lower-absorption results without SOC interaction disagrees with the experimental spectra (Supplementary Fig. 2). Conversely, in the results calculated with SOC, the absorption spectrum in the long-wavelength region can be reproduced qualitatively. The calculated lowest-energy absorption bands appeared at 813 nm (1.53 eV) and 777 nm (1.60 eV) for DX3 and DX2, respectively, which was consistent with the experimental results of 780 nm (1.58 eV) and 774 nm (1.60 eV) for the lowest-energy spin-forbidden bands that were allowed by SOC.

From DFT calculations, the assignment of each type of molecular orbitals (MOs) and its composition are shown in Fig. 2b–e and Supplementary Table 1, respectively. The lowest unoccupied MO is delocalized on the tricarboxy-terpyridine ligand. The highest occupied MO (HOMO) is mainly shared by the *d*-orbital (*d*_*yz*_) of the Ru atom, and the next HOMO (HOMO-1) and HOMO-2 are also shared by *d*_*xz*_ and *d*_*xy*_ orbitals of the Ru atom, respectively. The energy level of the HOMO-1 of DX3 is significantly destabilized as compared with that of DX1 (Supplementary Fig. 3). This is attributed to the ligand field splitting of the *d*_*xz*_-orbital of the Ru atom in DX3 being reduced due to overlapping with both the *d*_*xz*_-orbital of the Ru atom and the σ*-orbital of a phosphorus atom. The *π*-electron acceptor character of phosphine ligands depends on the steric effects^34^ and the electron affinity of the σ*-orbital^35^, therefore the dimethylphenylphosphine ligand on DX3 acts as stronger electron donor to Ru *d*_*xz*_-orbital than the methyldiphyenylphosphine ligand of DX2. The energy level of the HOMO-1 is destabilized in the order corresponding to DX1<DX2<DX3 (Supplementary Fig. 3).

According to the results of TD-DFT calculations based on perturbated SOC theory^36^ (PSOC-TD-DFT), the calculated spectrum was consistent with the SOC-TD-DFT calculation results (Supplementary Fig. 4). From the result of PSOC-TD-DFT, the triplet states (T1 and T2) and the singlet state (S1) of DX3 are mainly attributable to HOMO-1→LUMO and HOMO→LUMO, respectively (Supplementary Table 2). Supplementary Table 3 shows the contribution of the mixing of excited states by the PSOC effect; the relatively intense lower-energy band of DX3 (at 1.5381, eV) was ascribed to the mixed states that contain the excited S1 state and excited triplet states (T1 and T2). Recently, De Angelis and co-workers attributed the lower-energy absorption band of DX1 to the mixing of the S1 and T2 states by SOC^37^. In DX3, since the lower-energy level in the T2 triplet excited state is shifted close to the T1 state by destabilization of the HOMO-1 level (Supplementary Fig. 5), the excited T1 and T2 states have a similar electron distribution by configuration interaction. Accordingly, when the excited S1 state is mixed with the excited T1 and T2 states by SOC, DX3 indicated a red-shifted absorption band as compared with DX1 (Fig. 2f). On the basis of the perturbation theory, the oscillator strength of the singlet–triplet transition is inversely proportional to the energy difference between the singlet and triplet states^26^. In the case of DX3, since the energy differences between S1 and the triplet states (T1 and T2) increased by destabilization at the HOMO-1 level, the oscillator strength of the lower-absorption band decreased. This trend is consistent with the experimental results.

To analyse the S–T transition states of DX3, we performed ultrafast transient absorption (TA) spectroscopy. Supplementary Figure 6 shows the TA spectra of methyl-esterified ‘black dye'^30^ and methyl-esterified DX3 excited at 750 and 800 nm, respectively. The photoinduced absorption (PA) band in NIR area ∼1,000 nm of black dye is attributed to a broad triplet absorption (T1→T_*n*_) band^38^. Since esterified DX3 exhibited a similar PA band with esterified BD in NIR region, these PA bands in NIR part ∼1,000 nm can be also assigned to a triplet absorption. In the sub-ps time-resolved pump-probe measurement, the triplet population at 990 nm of esterified black dye is grown in ∼0.5 ps. It has been reported that the rising of the triplet population of black dye becomes relatively slow since it reflects the intersystem-crossing process from singlet to triplet excited states^39^. In contrast, we found out that the growth of the triplet PA band at 990 nm in esterified DX3 is completed within ∼0.3 ps. The results suggested that by exciting the esterified DX3 in NIR absorption band, the slow intersystem-crossing process was not observed, while the triplet PA band can be already probed. Considering the time resolution of the TA measurement of <250 fs, we concluded that the triplet excited states of esterified DX3 are generated directly by S–T transition.

The air-sensitive phosphorescence observed in both DX1, DX2 and DX3 at 77 K in DMF exhibited similar wavelength peaks at 920, 923 and 924 nm, respectively (Fig. 1d). The emission maxima were red shift at room temperature. The emission peaks observed with DX1, DX2 and DX3 at 298 K in DMF at 952, 969, 976 nm, respectively. The results suggested that the glass-to-fluid transition affects the Ru-P or Ru-N-bond length by rigidochromism^40^. The phosphorescence spectrum overlapped with the absorption spectrum in the low-energy band. Despite the fact that the phosphorescence of DX3 is a metal-to-ligand charge-transfer transition, DX3 showed a relatively small Stokes shift of 1,700 cm^−1^. The emission lifetime of the DX3 was 10 ns at 298 K, which is longer than 8 ns of DX1, and it had a sufficient lifetime relative to the electron injection rate. From differential pulse voltammetry of DX3 (Supplementary Fig. 7), the excited state of DX3 is expected to have a driving force comparable to that of DX1 for electron injection into the conduction band of TiO_2_.

### Single solar cell performance of DSSC and PSC

**Figure 3:**
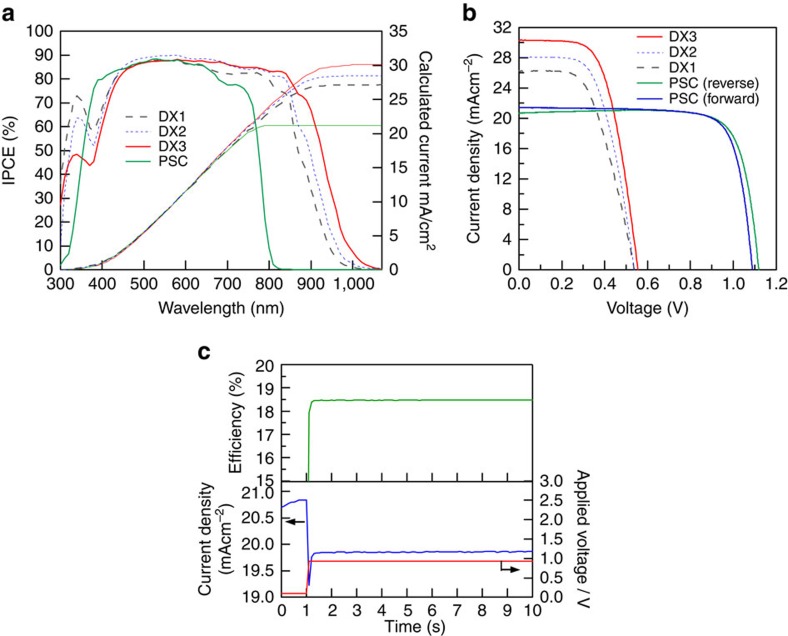
IPCE spectra and *J*–*V* characteristics of DSSCs and PSCs. (**a**) IPCE and integrated current spectra of DSSCs made of DX3, DX1 and PSC. (**b**) Current–voltage curves of the same devices shown in Fig. 2a under solar illumination (AM1.5G, 100 mW cm^−2^). (**c**) Photocurrent density (blue) and power conversion efficiency (green) as a function of time for the PSC held to the voltage (red) of the maximum output power point.

Figure 3a compares the incident photon-to-current efficiency (IPCE) spectra for the PSC to DSSCs using DX1, DX2 or DX3 as a sensitizer. While the PSC shows no photoresponse at wavelengths above 800 nm, the DX3-sensitized cell maintains a high IPCE values far beyond this limit. The IPCE exhibits a very high value—between 80 and 90% across a wide wavelength range between 400 and 850 nm—and reaches 70% at 900 nm with the tail extending to ∼1,100 nm. This is a remarkable advance over the DX1 sensitizer, which absorbs light only up to 1,000 nm. To date, DX3 achieves the best solar light-harvesting capacity of all known sensitizers approaching that of silicon solar cells. Remarkably, the DX3 achieves this performance with a thousand times smaller sensitizer quantity than silicon. Figure 3b shows three photocurrent density versus voltage (*J*–*V*) curves for DSSC employing DX1, DX2 or DX3 measured under standard sunlight (air mass (AM) 1.5 G at 100 mW cm^−2^). The device that used DX3 showed a high short-circuit current density (*J*_SC_) of 30.3 mA cm^−2^ and an open-circuit voltage (*V*_OC_) of 0.556 V, achieving an overall PCE of 10.2%. The photocurrent value of the DX3-sensitized cell is in agreement, within a 2% mismatch, with the calculated *J*_SC_ value from the overlap integration between the IPCE curve of DX3 and the AM1.5G spectral solar photon flux. This is the first time that a *J*_SC_ value over 30 mA cm^−2^ has been reached by a DSSC. The *V*_OC_ of 0.556 V is very respectable in view of its small optical bandgap of DX3 that amounts to 1.18 eV. The difference is 0.6 V, of which about 0.3 V represents the minimum voltage loss for a photovoltaic in AM1.5 sunlight^41^, while the remaining 0.3 eV is consumed to drive the electron injection from the excited DX3 into the TiO_2_ and its regeneration by the iodide electrolyte. This loss is much smaller than that for conventional ruthenium dyes. The difference is attributed to a reduction of intramolecular energy losses, as indicated by the small Stokes shift.

A histogram of 35 different devices made with DX3 is shown in Supplementary Fig. 8. The PCEs of DX3-sensitized cells are distributed in a range of 7–10%, the relatively low fill factor being caused by ohmic losses due to the high photocurrents. Optimizing the electric current collection will reduce the series resistance and enable a substantial increase in the PCE. The DX3-sensitized cell thus shows a large conversion efficiency with the additional advantage of achieving photoelectric conversion in the NIR above 1,000 nm (refs 21, 22, 23, 24, 25, 26, 27, 28, 29, 30).

Figure 3b shows the current–voltage characteristics of a perovskite solar cell (PSC) measured under standard conditions. The PSC showed a *V*_OC_ of 1.12 V, a *J*_SC_ of 20.7 mA cm^−2^, a fill factor of 0.794 and an overall efficiency of 18.4%. The *J*–*V* curve showed negligible hysteresis between the forward and the reverse sweep (Supplementary Table 4). The transient photocurrent response at the applied voltage of 0.93 V (voltage of the maximum power point) becomes constant within 100 ms, and the stabilized conversion efficiency of 18.4% was sustained at least for 60 s (Fig. 3c).

### Hybridized solar cell performance by a spectral splitting system

**Figure 4:**
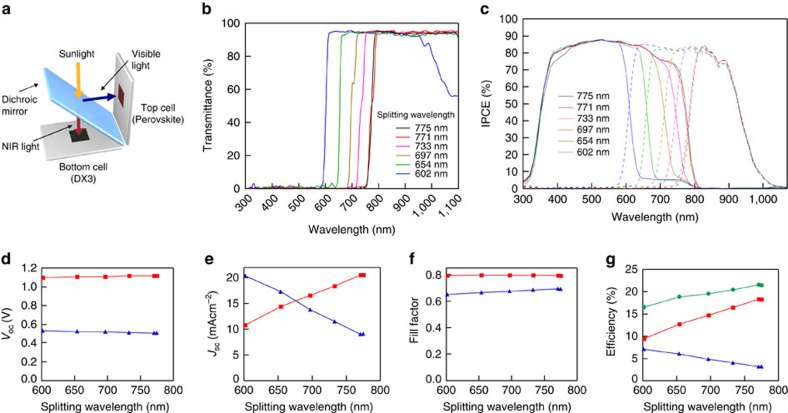
Device structure, IPCE spectra and *J*–*V* characteristics of the hybridized cell. (**a**) Device structure of the hybridized cell using a dichroic spectrum splitter. (**b**) Transmittance spectra of dichroic mirrors under measurement conditions such as incident of a 45° angle. (**c**) IPCE spectra of the individual components of the hybridized cell with various dichroic mirrors: visible absorbing cell, PSC (solid line); near-IR absorbing cell, DX3-based DSSC (dashed line). (**d**–**g**) Photovoltaic parameter and total power conversion efficiency dependence on the splitting wavelength of the dichroic mirrors: PSC (red), DX3-based DSSC (blue) and total power conversion efficiency of the hybridized cell (green).

The hybridized solar cell employing a spectral splitting system (Fig. 4a) was constructed using dichroic mirrors at an angle of 45° with splitting edge wavelengths of 602, 654, 697, 733, 771 and 775 nm (Fig. 4b). In the experiments, a DX3-sensitized cell with 9.4% efficiency was used as the NIR cell (Supplementary Fig. 9). The reproducibility of these results was verified independently at the KAST, Kanagawa, Japan. The DSSC showed a *V*_OC_ of 0.544 V, a *J*_SC_ of 29.6 mA cm^−2^, a fill factor of 0.591 and an overall efficiency of 9.53% (Supplementary Fig. 10). The results were achieved with a dual-source solar simulator (YSS-T150A Yamashita Denso, Japan), which has different lamp types combined (xenon and halogen lamps) to extend the spectrum far into the infrared. The spectral mismatch is <2% (class AAA) from ultraviolet to infrared, according to AM1.5G. The measurement was performed at 25±0.1 °C in a thermostat chamber, and the designated area of the cell was 0.1414, cm^2^. The results showed good agreement with the PCE measurements carried out in our own laboratory. The individual IPCE spectra (Fig. 4c) and the current–voltage characteristics of the visible and NIR light-harvesting cells measured under standard conditions are summarized in Fig. 4d–g and Supplementary Fig. 11. The IPCE spectra and the photocurrent value of the PSC were almost identical to the single cell at a splitting wavelength of longer than 771 nm. Figure 4g summarizes the total efficiency for the combination of the PSC and DSSC at each splitting wavelength. The PCE of the PSC increased as the splitting wavelength of the dichroic mirror gets longer, while the opposite trend was observed for the DSSC. The conversion efficiencies of the PSC reached a plateau at a splitting wavelength above 771 nm. At this wavelength, a maximum total PCE of 21.5% was achieved. Since the dichroic mirror divides the solar spectrum in two parts that are transmitted or reflected to each cell, the resistive losses in each component due to the photocurrent are reduced as compared with those of a single cell. The ohmic loss is proportional to *RI*^2^, where *R* is the series resistance in the inner cell and *I* is the photocurrent. Spectral splitting reduced the photocurrent of the DSSC component to about one-third the value measured at full sunlight. This reduces resistive losses and augments the fill factor. As a consequence, the PCE of an individual DX3-sensitized cell increased to 11.7% at 20 mW cm^−2^ simulated sunlight (Supplementary Table 5). This effect contributed to the high overall PCE of the hybridized cell.

## Discussion

The cell structure can be simplified by fabricating a series-connected mechanically stacked tandem solar cell. To realize the series connection, it is necessary to adjust the light absorption band so as to equal the photocurrent of each component under standard sunlight. As seen in Fig. 4e, the photocurrent value of each cell is matched at a splitting wavelength of ∼680 nm. If the splitting wavelength is shifted to 680 nm, as seen in Fig. 4g, the current-matched PCE would be expected to become about 19.5%. This PCE value is the same for the case of series-connected mechanically stacked tandem solar cell without any optical losses by the interlayer. In this case, the net voltage loss of lead halide PSC part increases up to ∼0.7 eV, since the lead halide PSC shows the *V*_OC_ of ∼1.1 V, whereas it absorbs the part of sunlight until 680 nm (1.82 eV). Using a high-voltage PSC with a lead bromide perovskite^42^,^43^, which shows the high-energy bandgap (∼2.3 eV), the series-connected tandem cell would achieve up to 22% PCE. In addition, the tandem cell can also be used for applications such as the photolysis of water^44^, since the *V*_OC_ of the tandem cell can achieve as high as 1.85 V with very high efficiency. Meanwhile, to realize an efficient series-connected tandem solar cell, it would be important to develop an efficient transparent PSC and/or develop the sensitizers that absorb the longer wavelength sunlight.

In summary, we have successfully constructed hybrid mesoscopic photovoltaics using a perovskite cell with a high *V*_OC_ and panchromatic DSSC by employing a broadband sensitizer, DX3, which we composed by molecular engineering based on triplet excited states. A single DX3-sensitized solar cell exhibited wide-ranging light absorption up to 1,100 nm and a very high photocurrent exceeding 30 mA cm^−2^. The PCE of the mesoscopic hybrid solar cell exceeds by a factor of two that of previously reported multijunction cells that employ organic photovoltaics. The performance of such solution-processed mesoscopic solar cells suggests that they have the potential to become an alternative to traditional inorganic semiconductor photovoltaics.

## Methods

### Synthesis of DX3

DX3 was synthesized as follows (the chemical reaction equations are shown in the Supplementary Methods): RuCl_3_ (TCI) was dissolved in dehydrated ethanol, and 4,4′-di-*tert*-butoxycarbonyl-4′′-methoxycarbonyl-2,2′;6′,2′′-terpyridine (methods for synthesis of the ligand are described in the Supplementary Methods) was then added. The reaction mixture was refluxed under argon for 6 h. The reaction mixture was cooled to room temperature, the solvent was then removed and the residue was dissolved into dry CHCl_3_ at 0 °C. NEt_3_ and dimethylphenylphosphine were then added to the reaction solution, and the reaction mixture was heated at 70 °C for 10 min. After cooling the reaction mixture, most of the solvent was removed under vacuum. The reaction residue was purified on a silica gel column chromatography using a mixed solvent (CHCl_3_:CH_3_CN=8.5: 1.5 v/v) as the eluent, and then the main band was collected. After removal of the solvent, a black powder was obtained. The black powder was then added to CF_3_COOH, and the reaction mixture was stirred for 30 min at room temperature. After removal of the solvent, the brown product was precipitated with diethyl ether, and the brown product was isolated by suction filtration and washed with diethyl ether. The brown powder was purified on a Sephadex LH-20 (GE Healthcare) column, using methanol as the eluent. The product was purified further by high-performance liquid chromatography system (Shimadzu) with high-performance liquid chromatography column (LiChrospher 100 DIOL, Merck). Yield 23%. ^1^H NMR (600 MHz, dimethyl sulphoxide (DMSO)-d_6_/D_2_O): δ 9.15 (s, 1H), 9.13 (s, 1H), 9.00 (s, 1H), 8.94 (s, 1H), 8.19 (d, *J*=7.8 Hz, 2H), 7.91 (m, 2H), 7.53 (m, 5H), 3.95 (s, 3H), 2.21 (d, *J*=7.4 Hz, 6H); ^13^C NMR (151 MHz, DMSO-d_6_/D_2_O): δ 164.9, 164.5, 160.7, 158.6, 157.8, 139.2, 138.8, 136.7, 134.6, 131.4, 130.1, 129.5, 125.6, 122.0, 120.8, 53.7, 14.4, 14.2; ^31^P NMR (243 MHz, DMSO-d_6_/D_2_O): δ 8.5; HRMS (*m*/*z*): [M-H]^−^ calculated for C_27_H_23_Cl_2_N_3_O_6_PRu, 687.9751; found, 687.9772.

### DSSC fabrication

A thin layer of TiO_2_ (Solaronix SA Ti-Nanoxide), containing a 24-μm thick film of 20-nm particles, with a 4-μm thick layer of 400-nm scattering particles was screen printed on an F-doped SnO_2_ (FTO) conducting glass with anti-reflective coating (Nippon Sheet Glass Co., Ltd, Tokyo; 10 Ω sq^−1^) using an automatic screen printer (Newlong Seimitsu Kogyo Co., Ltd, Tokyo) to guarantee repeatability. The prepared TiO_2_ electrodes were heated at 500 °C for 30 min under a dry atmosphere. The electrodes were placed in a TiCl_4_ aqueous solution for 30 min, after which the TiO_2_ electrodes were heated at 500 °C for 30 min in a dry condition (dew point −50 °C) for a total of five times. The temperatures at which the electrodes were immersed into a TiCl_4_ aqueous solution were 10 °C, 25 °C × 2 and 70 °C × 2 times. In the last heating step, the electrodes were allowed to cool to under 200 °C before dipping into the dye solution. The DX2 and DX3 dye solutions were prepared in DMF with a concentration of 7.5 × 10^−5^ M respectively, and the electrodes were left in the dye solutions for 12 h. A sandwich cell was prepared using the dye-anchored TiO_2_ film as a working electrode and a counter electrode, which was coated with platinum. The two electrodes were superimposed with a 30-μm spacer (DuPont-Mitsui Polychemicals Co., Ltd, Japan), and the superimposed electrodes were tightly held by clips. A thin layer of electrolyte solution containing 0.60 M 1,2-dimethyl-3-propylimidazolium iodide, 25 mM I_2_, 0.1 M LiI and 20 mM 4-*tert*-butylpyridine in a mixture of acetonitrile was introduced to the interelectrode space from the counter electrode side through a predrilled hole. Then, the drilled hole was sealed with a microscope cover slide.

### PSC fabrication

FTO (Pilkington, TEC8) substrate was cleaned in an ultrasonic bath containing detergents for 30 min, and then a dense blocking layer of TiO_2_ (60 nm, bl-TiO_2_) was deposited onto the FTO by spray pyrolysis using a 20-mM titanium diisopropoxide bis(acetylacetonate) solution (Aldrich) at 450 °C. A 100-nm thin mesoporous (mp) TiO_2_ was spin coated on top of the bl-TiO_2_/FTO substrate at 1,000 r.p.m. for 50 s using homemade TiO_2_ (∼50 nm) pastes. The pristine paste had been diluted in 2-methoxyethanol (1 g/5 ml), and calcinated at 500 °C for 1 h in air, which led to a thickness of about 100 nm. The (FAPbI_3_)_0.85_(MAPbBr_3_)_0.15_ perovskite solutions were then coated onto the mp-TiO_2_/bl-TiO_2_/FTO substrate heated to 50 °C by two consecutive spin-coating steps, at 1,000 and 5,000 r.p.m. for 5 and 10 s, respectively. During the second spin-coating step, 1 ml of ethyl ether was poured onto the substrate in accordance with previously reported procedures^8^,^45^. The 0.8 M solution for (FAPbI_3_)_0.85_(MAPbBr_3_)_0.15_ perovskite was obtained by dissolving (NH_2_)_2_CHI (FAI) and CH_3_NH_3_Br (MABr) with PbI_2_ and PbBr_2_ in (DMF) and DMSO (6: 1 v/v). The substrate was then dried on a hotplate at 100 °C for 10 min. A poly-triarylamine (EM index, Mn=17,500 g mol^−1^)/toluene (1 g/1 ml) solution with an additive of 7.5 μl of Li-bis(trifluoromethanesulphonyl) imide (Li-TFSI)/acetonitrile (170 mg/1 ml) and 7.5 μl of 4-*tert*-butylpyridine (TBP)/acetonitrile (1 ml/1 ml) was spin coated on a (FAPbI_3_)_0.85_(MAPbBr_3_)_0.15_/mp-TiO_2_/bl-TiO_2_/FTO substrate at 3,000 r.p.m. for 30 s. Finally, an Au counter electrode was deposited by thermal evaporation.

### Solar cell characterization

For photovoltaic measurement of the solar cells, the irradiation source was a 450-W xenon light source (YSS-80A; Yamashita Denso Co., Ltd, Japan), whose power of an AM1.5G, 100 mW cm^−2^ solar simulator was calibrated using a reference Si photodiode BS-520 (Bunkoukeiki Co., Ltd, Japan), which was calibrated by AK300 (Konica Minolta, Inc., Japan) at Kanagawa Academy of Science and Technology, Japan. The *J*–*V* curves were measured employing an HSV-110 (Hokuto Denko Co., Ltd, Japan) potentiostat. The scan rate was fixed at 10 mV s^−1^. Measurement of the IPCE was plotted as a function of the excitation wavelength using the incident light from a 150-W xenon lamp SM-250E (Bunkoukeiki Co., Ltd, Japan), which was focused through an SM-25 monochromator (Bunkoukeiki Co., Ltd, Japan). A light-shading mask was used on the photoanode of the solar cells to reduce scattered light from the edge of the glass electrodes (actual size: DSSC; 0.19 cm^2^, PSC; 0.16 cm^2^, designated area: single DSSC; 0.1414, cm^2^, single PSC and spectral splitting hybridized cell; 0.09 cm^2^). Dichroic mirrors (Semrock) were used as spectrum splitters. The light intensity and the spectral mismatch were measured using the grating spectroradiometer (LS-100, EKO Instruments Co., Ltd, Japan).

## Additional information

**How to cite this article:** Kinoshita, T. *et al.* Spectral splitting photovoltaics using perovskite and wideband dye-sensitized solar cells. *Nat. Commun.* 6:8834 doi: 10.1038/ncomms9834 (2015).

## Supplementary Material

Supplementary Information Supplementary Figures 1-11, Supplementary Tables 1-5, Supplementary Methods and Supplementary References.
