# Supplementary material for: Spectral splitting photovoltaics using perovskite and wideband dye-sensitized solar cells
Source: Nat Commun. 2015 Nov 5;6:8834. doi: 10.1038/ncomms9834 (PMC4667623; doi:10.1038/ncomms9834)
Supplement: Supplementary Information — Supplementary Figures 1-11, Supplementary Tables 1-5, Supplementary Methods and Supplementary References. [file ncomms9834-s1.pdf]

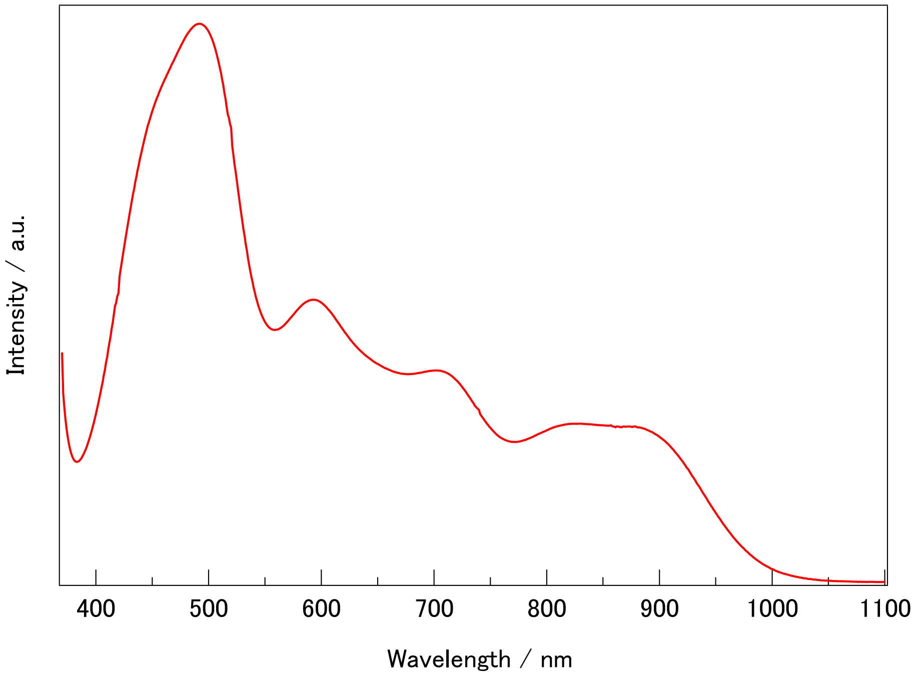

Supplementary Figure 1 Low-temperature absorption spectrum of esterfied-DX3 in toluene at 77K.

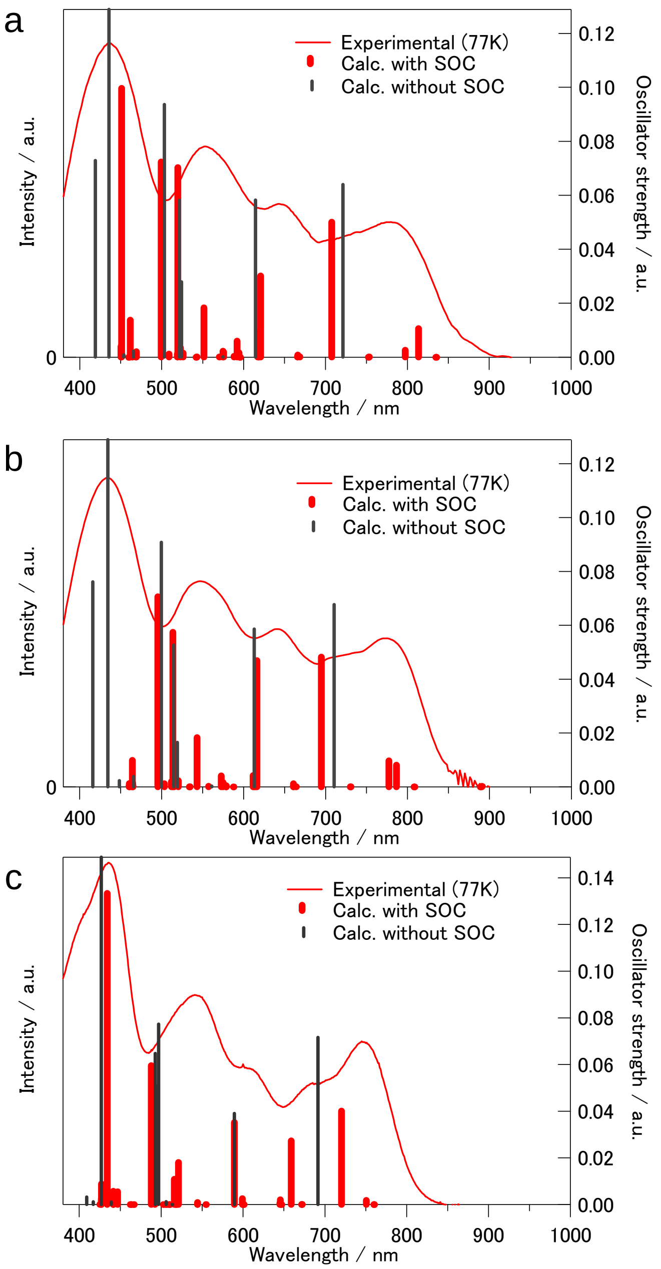

Supplementary Figure 2 Low temperature absorption spectra and calculated absorption spectra of DX3 (a), DX2 (b) and DX1 (c).

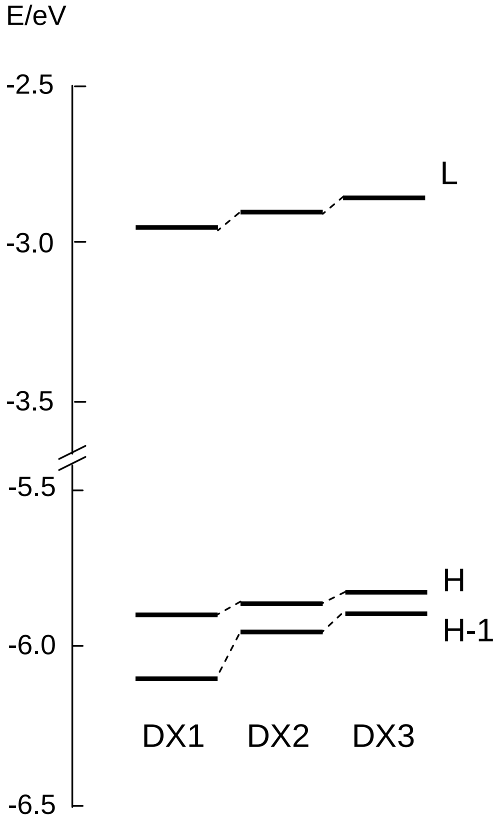

Supplementary Figure 3   Energy diagram of molecular orbitals for DX1, DX2 and DX3.

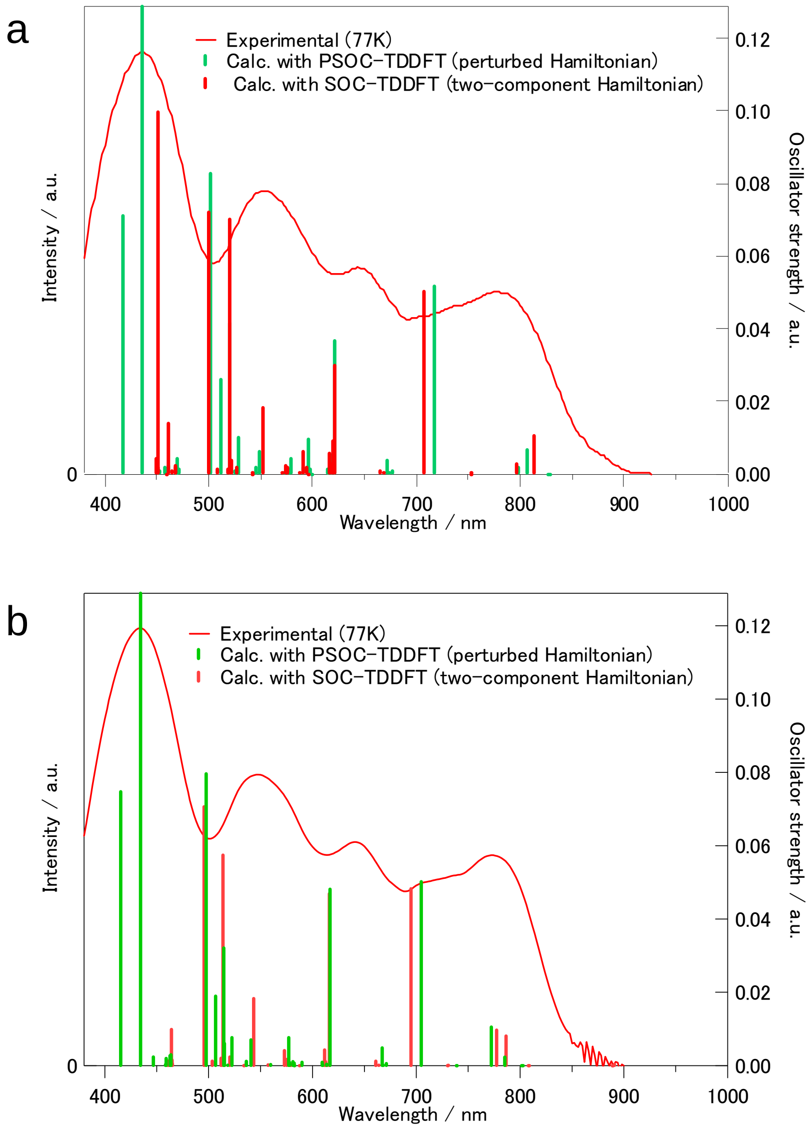

Supplementary Figure 4 Low-temperature absorption spectra and calculated absorption spectra of DX3 (a) and DX2 (b) with PSOC-TDDFT and SOC-TDDFT.

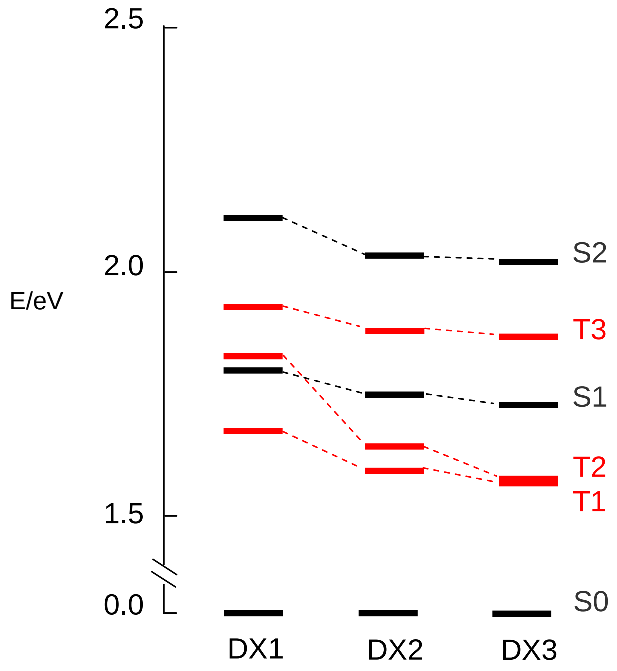

Supplementary Figure 5 Calculated excitated-states energy diagram of DX1, DX2 and DX3.

The energy level were calculated using scalar relativistic DFT at the M06/TZP using ADF program.

a

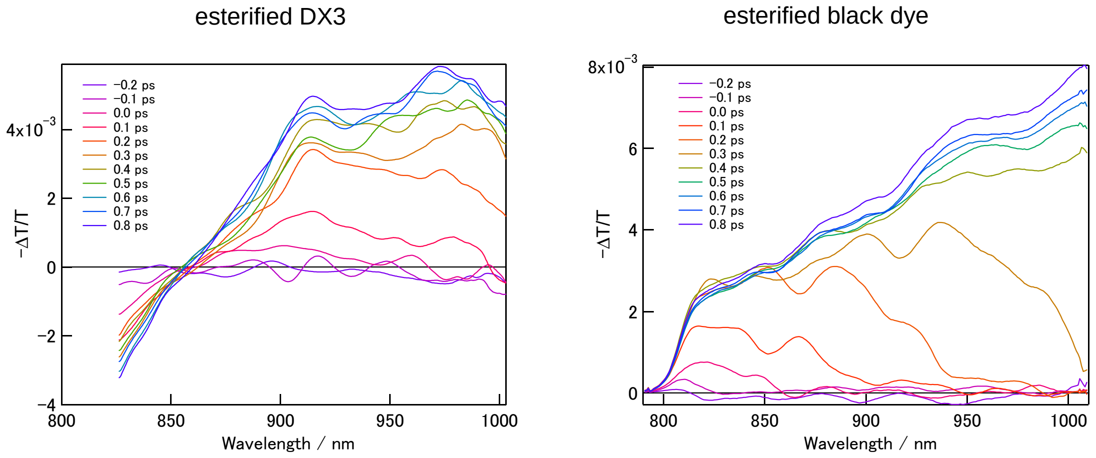

b

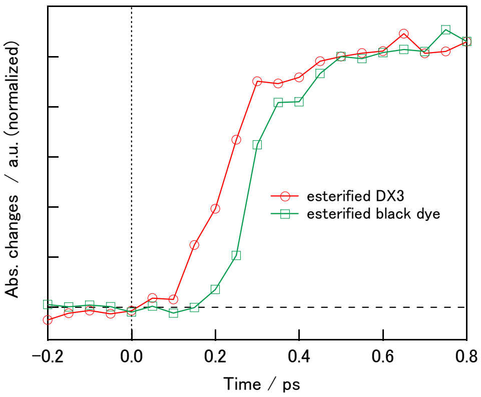

Supplementary Figure 6 Ultrafast transient absorption measurements of esterified DX3 and esterified black dye (a) Transient absorption spectra obtained upon sub-picosecond pump-and-probe of esterified DX3 (left) and esterified "black dye" (right) with several time delays between -0.2 ps and 0.8 ps with the pump wavelength at 800 nm (esterified DX3) and 750 nm (esterified black dye). (b) Transient absorption profiles with the prove wavelength at 990 nm taken with a temporal resolution of 50 fs, the spectra indicate the kinetics of the formation of the triplet excited state.

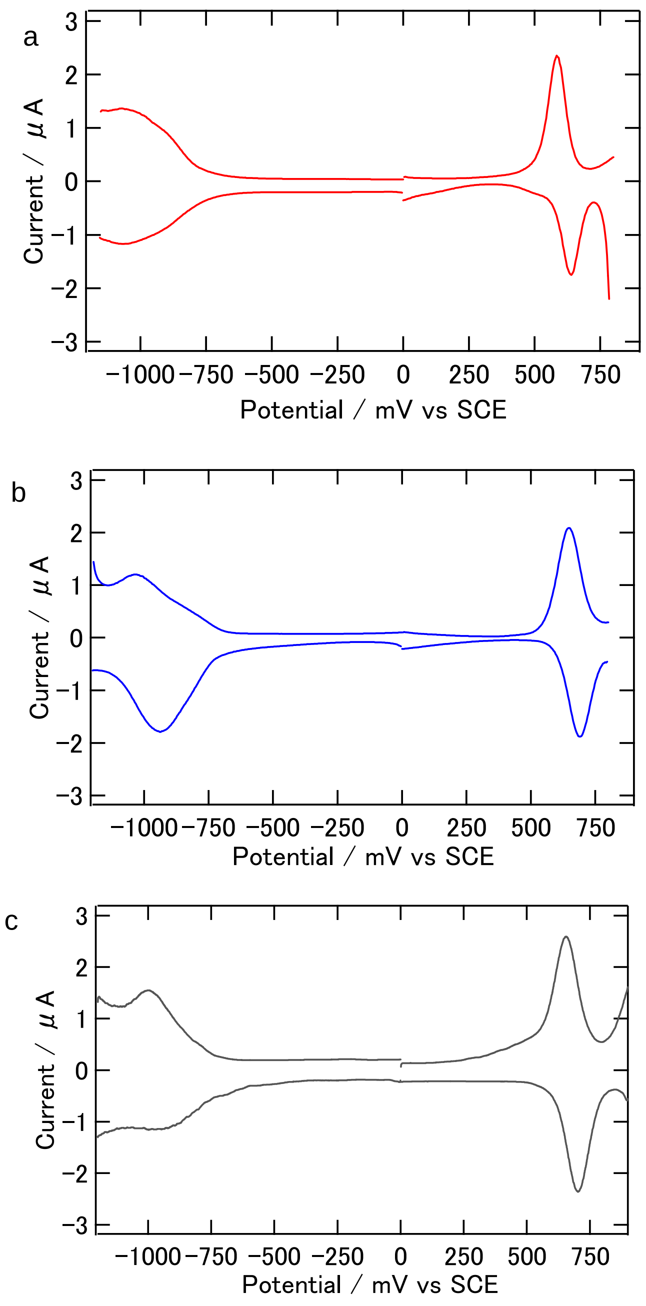

Supplementary Figure 7 Differential pulse voltammetry (DPV) of DX3 (a), DX2 (b) and DX1 (c). The redox potentials of DX3 were slightly shifted to negative values compared to DX1, and which were in good accordance with the small difference between the phosphorescence maxima of the two compounds of ~0.03 eV.

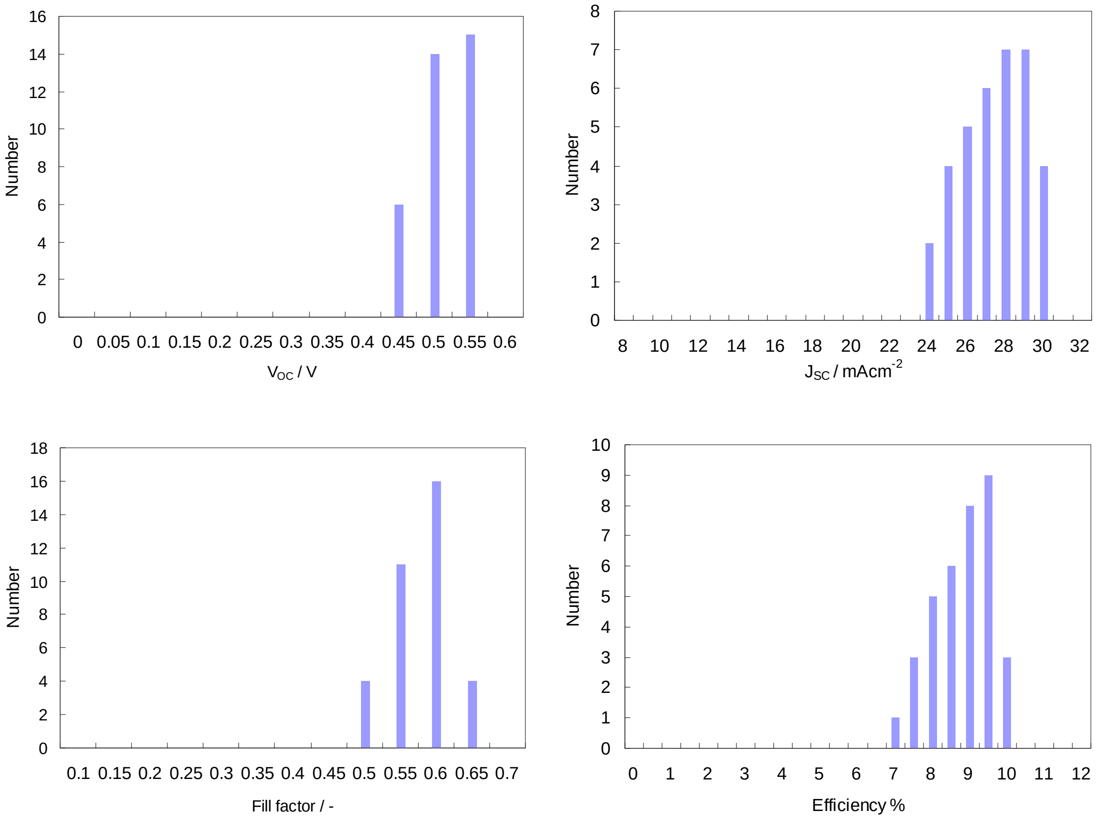

Supplementary Figure 8   Histogram of the device performance of DX3-sensitized cell.

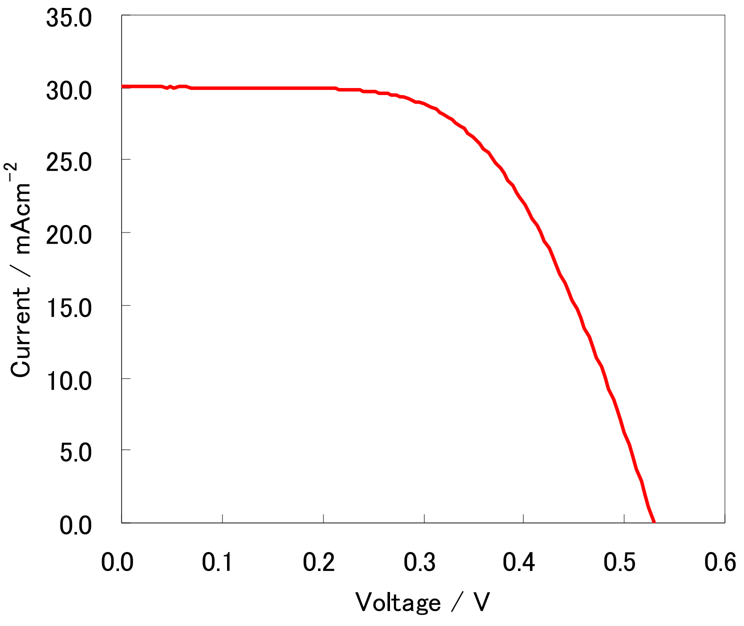

Supplementary Figure 9 Device performance of DX3-sensitized cell for double-junction solar cells. The DSSC showed a V_{OC} of 0.525 V, J_{SC} of 30.1 $mAcm^{-2}$, a fill factor (FF) of 0.595, and an overall efficiency of 9.4%.

I-V measurement

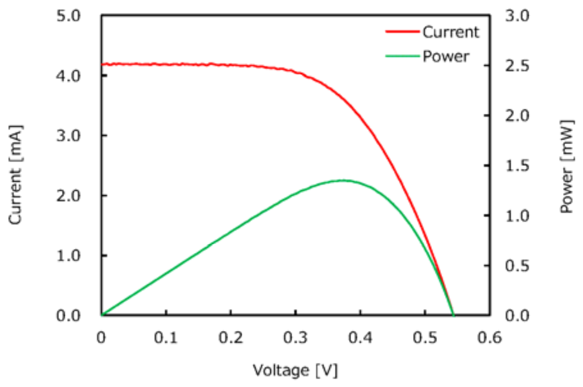

Date	2014/12/19
Sample No.	No.3
Designated Area	0.1414 cm^2
Measured Irradiance	100.0 mW/cm^2
Measured Temp.	25.1 ℃
Scan Mode	Isc to Voc
Sweep Time	25 s
Isc	4.190 mA
Voc	0.544 V
Pmax	1.347 mW
Ipmax	3.615 mA
Vpmax	0.373 V
F.F.	59.1%
Eff.	9.53%

Supplementary Figure 10 I-V measurement results of DX3 sensitized DSSC at external test center (Kanagawa Academy of Science and Technology (KAST), Kanagawa, Japan). The DSSC showed a V_{OC} of 0.544V, a J_{SC} of 29.6 $mAcm^{-2}$, a fill factor (FF) of 0.591, and an overall efficiency of 9.53%.

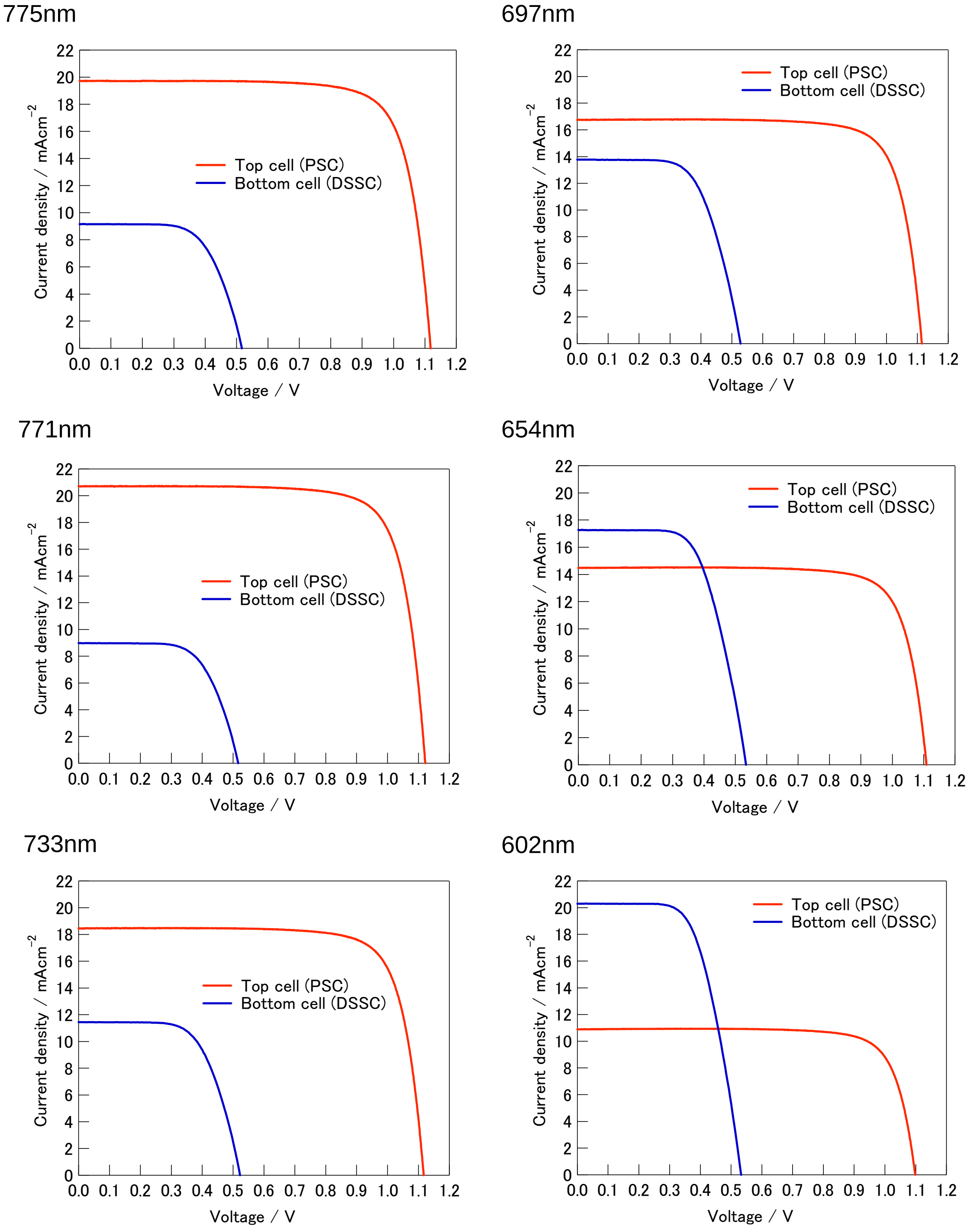

Supplementary Figure 11 J-V characteristics of each component cell of hybridized solar cells with various dichroic mirrors.

Supplementary Table 1 Molecular orbitals with dominant character (%) for DX3

	Contribution / % (>10%)
HOMO	61 (Ru:d_{yz}), 18 (Cl)
H−1	55 (Ru:d_{xz}), 21 (Cl)
H−2	59 (Ru:d_{xy}), 15 (Ru:d_{z^2})

Supplementary Table 2 Excitation characteristics of DX3.

State	E/eV	f	MO contributiuon (%)							
S1	1.720	0.064	H	->	L	(98)				
S2	2.018	0.058	H-1	->	L	(84)	H	->	L+1	(14)
T1	1.565	0	H	->	L	(64)	H-1	->	L	(32)
T2	1.574	0	H-1	->	L	(63)	H	->	L	(33)

The H, H-1, L and L+1 in the character column denote the HOMO, HOMO-1, LUMO, and LUMO+1, respectively.

Supplementary Table 3 Excitation states characteristics of complex DX3 with the perturbated SOC effect.

State	E/eV	f	Contribution (%)
1	1.4972	0.0000	T1 (43) T2 (40)
2	1.4992	0.0000	T1 (53) T2 (46)
3	1.5381	0.0068	T1 (58) T2 (28) S1 (10)
4	1.5523	0.0018	T2 (61) T1 (34)
5	1.6267	0.0000	T2 (49) T1 (46)
6	1.6268	0.0000	T2 (54) T1 (46)
7	1.7281	0.0517	S1 (81) T2 (10)
8	1.8337	0.0007	T3 (89)
9	1.8426	0.0001	T3 (90)
10	1.8446	0.0035	T3 (86)

Supplementary Table 4 Influence of scanning conditions on PSC performances.

Direction	V_{OC} /V	J_{SC} /mAcm^{-2}	FF	Eff.
Rev.	1.12	20.7	0.794	18.4
Fwd.	1.09	21.4	0.779	18.2

Reverse scanning (Rev.) is from open-circuit to short-circuit under the forward bias voltage, and forward scanning (Fwd.) is from short-circuit to open-circuit under the forward bias voltage.

Supplementary Table 5 Photovoltaic parameters of the DX3-sensitized cell under different incident light intensities.

int. / $mWcm^{-2}$	V_{OC} /V	J_{SC} /$mAcm^{-2}$	FF	Eff.
100	0.552	30.3	0.60	10.0
59	0.524	18.7	0.64	10.7
45.9	0.516	14.7	0.66	10.9
35.5	0.512	11.4	0.67	11.0
27.8	0.508	8.97	0.69	11.3
20.9	0.504	6.98	0.69	11.7
15.9	0.496	5.03	0.70	10.9
12.6	0.488	3.95	0.71	10.9
7.4	0.472	2.21	0.72	10.1

Supplementary Methods

1. Measurements methods

UV-vis absorption spectra were measured using a JASCO V-570 spectrometer. The steady-state emission and time-resolved emission spectra were measured by exciting the sample with a pulse from an active modelocked Nd:YAG laser, using the frequency doubled line at 532 nm. The emitted light was detected with a Hamamatsu R5509-73 photomultiplier operated in single-photon counting mode. The emission lifetimes were measured using a time-correlated single photon counting system (Hamamatsu C7990). The emission lifetime data were analyzed using the deconvolution method with an instrumental response function (IRF). A Dewar vessel was used for the measurements at 77K. Ultrafast transient absorption spectra were measured by the pump-probe method. Seed pulses are generated using an amplified Ti:sapphire laser setup (Spectra Physics Hurricane), which provides 130 fs laser pulses at 800 nm at a repetition rate of 1.0 kHz. The pump light (wavelength at 800 nm or 750 nm) was generated using a optical parametric amplifiers (Topas, Light Conversion). The probe was a white light continuum pulse generated in a thin sapphire crystal (thickness 3mm). ^{1}H NMR, ^{13}C NMR and ^{31}P NMR spectra were recorded with a Bruker Avance 600 MHz NMR spectrometer. Electrochemical data were obtained by differential pulse voltammetry using a three-electrode cell and a BAS100B/W electrochemical analyzer. The counter electrode and the

working electrode were platinum electrodes, and the reference electrode was a saturated calomel electrode (SCE), and the supporting electrolyte was 0.1 M tetrabutylammonium hexafluorophosphate.

2. Computation Methods

The geometry optimizations were performed by the *Gaussian 09* program[(1)] using DFT with the M06 meta-hybrid exchange–correlation functional. The geometry optimizations were performed in ethanol solution using "Triple-ζ" quality basis sets were employed for the ligands (6-311G*) [(2)] and the Ru (LanL2DZ) [(3)]. A relativistic effective core potential (ECP)[(3)] on Ru replaced the inner core electrons leaving the outer core $[(4s)^2(4p)^6]$ electrons and the $(4d)^6$ valence electrons of Ru(II). The geometries were fully optimized without symmetry constraints. Solvation effects were included by means of the conductor-like polarizable continuum model (C-PCM) [(4)] with ethanol parameters.

TD-DFT [(5)] excited states calculations were performed by the Amsterdam density functional program package [(6)] (ADF2013.01c) based on the zero-order regular approximation (ZORA) [(7)] two-component relativistic Hamiltonian. The M06 functional was used with all electron Slater-type orbital basis sets: triple-zeta + polarization (TZP). The ZORA Hamiltonian is the zeroth order regular approximation to the Dirac Hamiltonian. The ZORA Hamiltonian includes a scalar relativistic (SR) and a SOC part.

$$H^{ZORA} = H^{SR} + H^{SOC}$$

In SOC-TDDFT, SOC part is fully included self-consistently during the Self-Consistent Field (SCF) and TDDFT calculations. In contrast, PSOC-TDDFT is included the SOC effect as a perturbation for SR Hamiltonian. SOC-TDDFT is a more theoretically reasonable method, however it is more computationally demanding than PSOC-TDDFT for the same conditions. PSOC-TDDFT gives the correlation of the SOC effects for each excited state qualitatively. In this study, we performed the SOC-TDDFT calculations which included 40 spin mixed excitations. In PSOC-TDDFT, 10 singlet + 10 triplet excitations were calculated using the TDDFT calculations with SR Hamiltonian, which are used as the basis for the perturbative expansion. Solvation effects were included via COSMO continuum model [(8)] using ethanol parameters.

3. Materials

All organic solvents used were of puriss grade from Wako Pure Chemical Industries, Ltd, Japan. DMF was distilled with BaO in vacuum, and treatment with molecular sieves 3A for 1day. After deaeration the solvent was stored under Ar and used as soon as possible.

4. Synthesis and characterizations

Synthetic scheme for DX2 and DX3

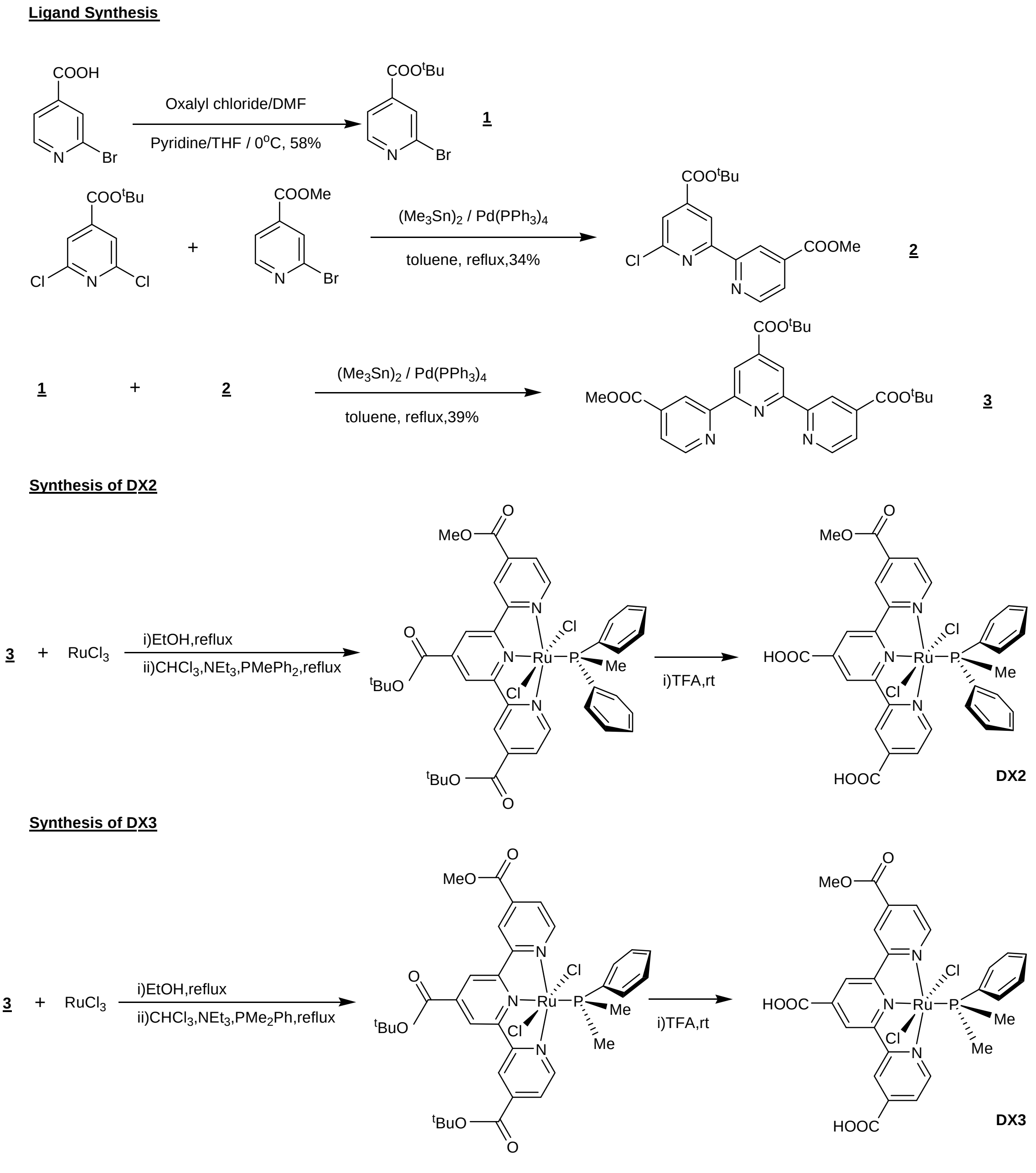

Synthesis of *tert*-butyl-2-bromoisonicotinate (1).

2-bromoisonicotinate (TCI) (2.5 g, 12.38 mmol) was added into dry THF (20 ml), pyridine (5 ml)

and DMF (small quantity) under Ar atmosphere, and then oxalyl chloride (TCI) (3.2 ml, 37.14 mmol) was dropped slowly at 0°C in an ice bath. The reaction solution was stirred for 3h at room temperature, and then the solvent was removed under vacuum. The reaction residue was then added dry THF (10 ml) and dry CH_2Cl_2 (20 ml), and the reaction solution was then added slowly tBuOK in THF solution (2.78 g, 24.76 mmol) at 0°C. The reaction mixture was then stirred overnight at room temperature.

The reaction solution was then quenched by addition of water in an ice bath, the reaction mixture was then extracted with water and CH_2Cl_2, and the organic layer was then washed with $NaHCO_3$ (aq) and then the organic layer was dried with Na_2SO_4. After removal of Na_2SO_4, the solvent was evaporated, the reaction mixture was then purified using column chromatography on silica gel ($CHCl_3$ NEt_3 = 100:1 v / v). Yield 58 % (1.85 g, 7.17 mmol). ^{1}H-NMR (500MHz, $CDCl_3$) : δ 8.51 (d, J=5.0 Hz, 1H), 7.81 (s, 1H), 7.72 (d, J=5.0 Hz, 1H), 1.60 (s, 9H); ^{13}C NMR (151 MHz, $CDCl_3$): δ 163.0, 151.1, 143.0, 142.1, 128.1, 83.6, 28.4; HRMS (m/z): $[M+H]^+$ calcd. for $C_{10}H_{13}BrNO_2$, 258.0124; found, 258.0132.

Synthesis of 6-Chloro-4’-methylcarbonyl-4-*tert*-butoxycarbonyl-2,2'-bipyridine (2).

Methyl-2-bromoisonicotinate (TCI) (920 mg, 4.26mmol), hexamethylditin (Wako) (1.395 g, 912.2 μl, 4.26 mmol) and tetrakis(triphenylphosphine)palladium(0) (TCI) (246 mg, 0.213 mmol, 5

mol%) were added into dry toluene (25ml), the reaction mixture was refluxed for 3h under Ar. After cooling the reaction mixture, tetrakis(triphenylphosphine)palladium(0) (246 mg, 0.213 mmol, 5 mol%) and *tert*-butyl-2,6-dicholroisonicotinate (TCI) (2.643 g, 10.65 mmol) were then added into the reaction mixture. The reaction mixture was then refluxed for 20 under Ar, after which the solution was filtered and evaporated under vacuum to afford a brown residue. The brown product was precipitated with MeOH. The residue was purified by flash chromatography on silica gel ($CHCl_3$:EtOAc=50:1 v/v as eluent). Yield 519 mg, 35%. ^{1}H-NMR (500MHz, $CDCl_3$) : δ 8.91 (s, 1H), 8.86 (d, J=3.9 Hz, 1H), 8.81 (s, 1H), 7.92 (d, J=3.9 Hz, 1H), 7.87 (s, 1H), 4.02 (s, 3H), 1.64 (s, 9H); ^{13}C NMR (151 MHz, $CDCl_3$): δ 165.9, 163.2, 157.1, 155.6, 152.1, 151.1, 150.5, 143.7, 139.0, 124.6, 124.0, 121.1, 119.6, 83.6, 53.2, 28.4; HRMS (m/z): $[M+H]^+$ calcd. for $C_{17}H_{18}ClN_2O_4$, 349.0950; found, 349.0936.

Synthesis of 4,4'-di-*tert*-butoxycarbonyl-4”-methoxycarbonyl -2,2';6',2''-terpyridine (3).

To a solution of *tert*-butyl-2-bromoisonicotinate (1) (369.1 mg, 1.43 mmol) and hexamethylditin (468.5 mg, 306.2 µl, 1.43 mmol) in toluene (10ml) was added tetrakis(triphenylphosphine)palladium(0) (99 mg, 0.0858 mmol, 6 mol%). The resulting solution was refluxed for 3 h under Ar. After cooling the reaction mixture, tetrakis(triphenylphosphine)palladium(0) (99 mg, 0.0858 mmol, 6 mol%) and

6-Chloro-4’-methylcarbonyl-4-tert-butoxycarbonyl-2,2'-bipyridine (2) (498.8 mg, 1.43mmol) were then added into the reaction mixture. The reaction mixture was then refluxed for 20 under Ar, after which the solution was filtered and evaporated under vacuum to afford a brown residue. The brown product was precipitated with n-hexane. The residue was purified by gel-chromatography (GPC) (eluent used was $CHCl_3$). Yield 39 % (275.65 mg, 0.561 mmol). ^{1}H-NMR (500MHz, $CDCl_3$) : δ 9.12-9.09 (m, 2H), 8.96 (s, 2H), 8.90-8.86 (m, 2H), 7.93-7.85 (m, 2H), 4.02 (s, 3H), 1.69 (s, 9H), 1.67 (s, 9H); ^{13}C NMR (151 MHz, $CDCl_3$): δ 166.1, 164.6, 157.0, 156.7, 156.4, 156.2, 150.4, 142.3, 140.8, 138.9, 124.7, 123.6, 121.3, 121.0, 82.9, 53.2, 28.5; HRMS (m/z): $[M+H]^+$ calcd. for $C_{27}H_{30}N_3O_6$, 492.2129; found, 492.2135.

Synthesis of DX2.

$RuCl_3$ (TCI) was dissolved in dehydrated ethanol, and 4,4'-di-tert-butoxycarbonyl-4”-methoxycarbonyl -2,2';6',2"-terpyridine (3) was then added. The reaction mixture was refluxed under argon for 6 h. The reaction mixture was cooled to room temperature, the solvent was then removed, and the residue was dissolved into dry $CHCl_3$ at 0˚C. NEt_3 and methyldiphenylphosphine were then added to the reaction solution, and the reaction mixture was heated at 70˚C for 10 min. After cooling the reaction mixture, most of the solvent was removed under vacuum. The reaction residue was purified on a silica gel column chromatography

using a mixed solvent ($CHCl_3$:AN=9:1 v/v) as the eluent, and then the main band was collected. After removal of the solvent, a black powder was obtained. The black powder was then added to CF_3COOH, and the reaction mixture was stirred for 30 min at room temperature. After removal of the solvent, the brown product was precipitated with diethyl ether, and the brown product was isolated by suction filtration and washed with diethyl ether. The brown powder was purified on a Sephadex LH-20 (GE Healthcare) column, using methanol as the eluent. The product was purified further by HPLC system (Shimadzu, Japan) with HPLC column (LiChrospher 100 DIOL, Merck, Germany). Yield 22%. ^{1}H NMR (600 MHz, DMSO-d_6/D_2O): δ 9.15 (s, 1H), 9.12 (s, 1H), 9.00 (s, 1H), 8.95 (s, 1H), 8.19 (d, J = 7.7 Hz, 2H), 8.01 (m, 4H), 7.43 (m, 8H), 3.94 (s, 3H), 2.41 (d, J = 6.4 Hz, 3H); ^{13}C NMR (151 MHz, DMSO-d_6/D_2O): δ 164.9, 164.5, 160.7, 158.7, 157.5, 137.2, 137.0, 136.9, 135.3, 133.7, 130.2, 129.0, 125.5, 122.0, 120.9, 53.6, 14.8, 13.9; ^{31}P NMR (243 MHz, DMSO-d_6/D_2O): δ 17.2; HRMS (m/z): $[M-H]^-$ calcd. for $C_{32}H_{25}Cl_2N_3O_6PRu$, 749.9907; found, 749.9932.

Supplementary References

(1) Frisch, M. J., *et al. Gaussian 09*, revision A.02; Gaussian, Inc.:Wallingford, CT, 2009.

(2) Krishnan, R., Binkley, J. S., Seeger, R. & Pople, J. A. Self-consistent molecular orbital methods. XX. A basis set for correlated wave functions. *J. Chem. Phys.* **72,** 650-654 (1980).

(3) Hay, P. J., Wadt, W. R. Ab initio effective core potentials for molecular calculations. potentials for the transition metal atoms Sc to Hg. *J. Chem. Phys*. **82**, 270-283 (1985)

(4) Cossi, M., Rega, N., Scalmani, G.; Barone, V. Energies, structures, and electronic properties of molecules in solution with the C-PCM solvation model. *J. Comput. Chem.* **24,** 669-681 (2003)

(5) Bauernschmitt, R. & Ahlrichs, R. Treatment of electronic excitations within the adiabatic approximation of time dependent density functional theory. *Chem. Phys. Lett.* **256,** 454-464 (1996)

(6) E. J. Baerends, *et al*., ADF2013.01c, SCM, Theoretical Chemistry, Vrije Universiteit, Amsterdam, The Netherlands, http://www.scm.com

(7) van Lenthe, E., Baerends, E. J. & Snijders, J. G. Relativistic total energy using regular approximations. *J. Chem. Phys.* **101**, 9783-9792 (1994).

(8) Pye, C. C. & Ziegler, T. An implementation of the conductor-like screening model of solvation within the Amsterdam density functional package. *Theor. Chem. Acc.* **101**, 396–408 (1999).
